# Therapeutic Validation of Venous Pulsatile Tinnitus and Biomaterial Applications for Temporal Bone Reconstruction Surgery Using Multi-sensing Platforms and Coupled Computational Techniques

**DOI:** 10.3389/fbioe.2021.777648

**Published:** 2022-01-03

**Authors:** Yue-Lin Hsieh, Xiuli Gao, Xing Wang, Fu-Chou Hsiang, Xinbo Sun, Wuqing Wang

**Affiliations:** ^1^ Department of Otology and Skull Base Surgery, Eye Ear Nose & Throat Hospital, Fudan University, Shanghai, China; ^2^ NHC Key Laboratory of Hearing Medicine, Shanghai, China; ^3^ Department of Radiology, Eye Ear Nose & Throat Hospital, Fudan University, Shanghai, China; ^4^ School of Mechanical and Automotive Engineering, Xiamen University of Technology, Xiamen, China; ^5^ Department of Orthopedics, Shanghai JiaoTong University Affiliated Sixth People’s Hospital, Shanghai, China; ^6^ BOACH Acoustic Laboratory, BOACH Acoustic Technology Co., Ltd., Xianyang, China

**Keywords:** pulsatile tinnitus, reconstructive surgery, dehiscence, temporal bone, materials, acoustic sensor, displacement sensor, transmission pathway

## Abstract

The application of grafts and biomaterials is a cardinal therapeutic procedure to resolve venous pulsatile tinnitus (PT) caused by temporal bone dehiscence during transtemporal reconstructive surgery. However, the transmission mechanism of venous PT remains unclear, and the sound absorption and insulation properties of different repair materials have not been specified. This study quantifies the vibroacoustic characteristics of PT, sources the major transmission pathway of PT, and verifies the therapeutic effect of different material applications using joint multi-sensing platforms and coupled computational fluid dynamics (CFD) techniques. The *in vivo* intraoperative acoustic and vibroacoustic characteristics of intrasinus blood flow motion and dehiscent sigmoid plate of a typical venous PT patient were investigated using acoustic and displacement sensors. The acoustical, morphological, and mechanical properties of the dehiscent sigmoid plate, grafts harvested from a cadaveric head, and other biomaterials were acquired using acoustical impedance tubes, micro-CT, scanning electron microscopy, and mercury porosimetry, as appropriate. To analyze the therapeutic effect of our previous reconstructive techniques, coupled CFD simulations were performed using the acquired mechanical properties of biomaterials and patient-specific radiologic data. The peak *in vivo* intraoperatively gauged, peak simulated vibroacoustic and peak simulated hydroacoustic amplitude of PT prior to sigmoid plate reconstruction were 64.0, 70.4, and 72.8 dB, respectively. After the solidified gelatin sponge–bone wax repair technique, the intraoperative gauged peak amplitude of PT was reduced from 64.0 to 47.3 dB. Among three different reconstructive techniques based on CFD results, the vibroacoustic and hydroacoustic sounds were reduced to 65.9 and 68.6 dB (temporalis–cartilage technique), 63.5 and 63.1 dB (solidified gelatin sponge technique), and 42.4 and 39.2 dB (solidified gelatin sponge–bone wax technique). In conclusion, the current novel biosensing applications and coupled CFD techniques indicate that the sensation of PT correlates with the motion and impact from venous flow, causing vibroacoustic and hydroacoustic sources that transmit via the air-conduction transmission pathway. The transtemporal reconstructive surgical efficacy depends on the established areal density of applied grafts and/or biomaterials, in which the total transmission loss of PT should surpass the amplitude of the measured loudness of PT.

## Introduction

Vascular pulsatile tinnitus (PT) is an abnormal self-perception of pulse-synchronous vascular somatosound (Kim et al., 2016). Venous PT comprises the largest demographic of all vascular types of PT (Mattox and Hudgins, 2008). Venous PT is subcategorized under objective tinnitus, whereby the sensation of PT immediately abates or disappears when the outflow at the upper jugular vein is halted (Mattox and Hudgins, 2008; Eisenman et al., 2018). The most common finding among patients with venous PT is sigmoid sinus wall anomalies, i.e., sigmoid sinus wall dehiscence and sigmoid sinus diverticulum (Dong et al., 2016). In recent years, much attention has been focused on clinical characteristics and the transtemporal management of venous PT caused by sigmoid sinus wall anomalies (Guo and Wang, 2015; Zeng et al., 2016; Eisenman et al., 2018; Hsieh and Wang, 2020; Lee et al., 2020).

Although the cause of venous PT remains unknown, studies have extrapolated that the gradual formation of sigmoid plate dehiscence and increased flow kinetic energy may be closely related to the increase in PT (Eisenman, 2011). The structural deformity of the sigmoid plate overlying the dural venous sinus vessel has been postulated to develop under long-term transverse sinus flow anomalies or increased intracranial pressure (Hewes et al., 2020; Mu et al., 2020; Hsieh Y. L. et al., 2021). As dehiscence forms, the sound wave originating from the intrasinus vascular flow is hypothesized to permeate through the thinned/dehiscent bony plate, reaching the inner ear through subspaces of the mastoid bone via the air-conduction transmission pathway (Eisenman, 2011; Bhatnagar et al., 2020). However, some authors have reported the vibroacoustic generation of PT due to displacement of the vascular wall as a causative factor of PT (Tian et al., 2019; Mu et al., 2021). Despite the dichotomous view of PT acoustic origination, both hypotheses have postulated that PT is transmitted by air, and the bone-conduction transmittance is considered less significant (Eisenman et al., 2018; Bhatnagar et al., 2020).

Commonly used sound-impeding/absorbing repair materials (e.g., autologous materials: bone pate/plate, postauricular cartilage, and temporalis muscle; applied biomaterials such as gelatin sponge, bone cement, and bone wax) have been topically applied. Surgical glues and other special materials (e.g., acellular dermis) over the dehiscent sigmoid plate have been proven to prevent sound from reaching the cochlea (Guo and Wang, 2015; Wang et al., 2017; Eisenman et al., 2018; Hsieh and Wang, 2020; Lee et al., 2020). These biomaterials are often layered and fixated to the surface of the vascular wall dehiscence adjacent to the inner ear to abolish PT (Liu et al., 2019). According to one systematic review pertaining to this subject, autologous bone chips and temporalis fascia are the most commonly used materials for transtemporal reconstructive surgery, but these result in a high rate of surgical failure (Wang et al., 2017). Despite the practice of multiple resurfacing methods, the volume of sound insulation and mechanical characteristics of each biomaterial for temporal bone reconstruction have not been documented hitherto.

Extrinsic sensing applications to assess the sound transmission of PT inside the mastoid bone have recently been attempted on both *in vivo* and *in vitro* 3D-printing platforms (Tian et al., 2019; Valluru et al., 2020; Hsieh and Wang, 2021). As the sound wave pressure dampens due to surrounding human anatomical structures, the acquisition of the absolute amplitude of PT *in vivo* can be rather arduous. However, the coupled computational fluid dynamics (CFD) technique has recently succeeded in revealing hydroacoustic and flow fluid–structure interaction details of PT but requires accurate establishment of boundary conditions (Tian et al., 2017; Hsieh Y.-L. et al., 2021; Mu et al., 2021). Therefore, continuous refinements on applying *in vivo* sensed mechanical characteristics of realistic anatomical structures and biomaterials are warranted, to support computational simulations and quantitatively verify surgical efficacy.

The therapeutic effect and sound insulation properties of the applied grafts/biomaterials have not been ascertained. Since the application of sound-insulating materials is a key procedure in transtemporal reconstructive surgeries to resolve PT, this study aims (1) to discover and quantify the origin and the airborne acoustic profile of PT and (2) to validate the therapeutic effect after the application of biomaterials combining *in vivo*, *in vitro*, and computational outcomes. Knowledge of the source and transmission pathways of PT and sound insulation characteristics of different biomaterials should aid surgeons in enhancing transtemporal surgical efficacy, providing a biomechanical basis for PT caused by dehiscence of the temporal bone.

## Materials and Methods

### Clinical Data and Study Design

**FIGURE 1:**
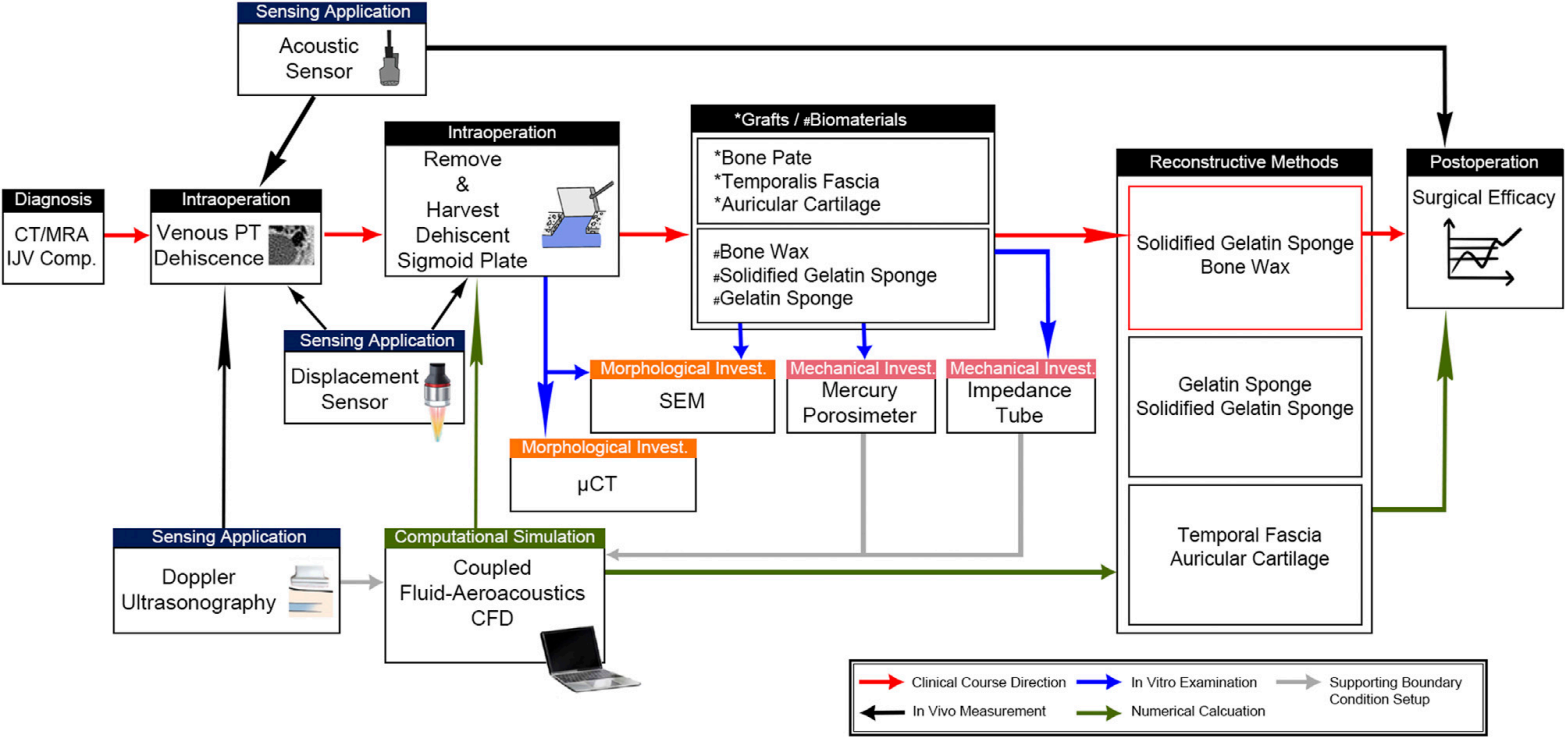
Complete workflow to determine the source of PT and therapeutic efficacy of graft/biomaterial applications. Intraoperative sensing applications were deployed to differentiate the intrasinus flow-borne and/or solid-borne vibration characteristics. Afterward, the morphological and mechanical properties of harvested dehiscent sigmoid plate and cadaveric grafts/external biomaterials were studied using μCT, SEM, mercury porosimetry, and impedance tubes. These mechanical properties were acquired to support the coupled fluid-aeroacoustics CFD simulation for the quantification of the surgical efficacy of the three reconstructive techniques. CFD data were also verified by the acoustic sensing results to justify the aeroacoustics amplitude of PT.

This study recruited a female patient with venous PT whose PT resolved after sigmoid sinus wall reconstruction surgery in January 2021. The surgical technique has been previously described (Guo and Wang, 2015; Hsieh and Wang, 2020; Hsieh Y. L. et al., 2021). Her PT was deemed eliminable during the ipsilateral internal jugular vein compression and water occlusion tests. The patient was diagnosed with PT caused by a dehiscent sigmoid plate after acquiring temporal bone computed tomography (CT) (Siemens AG, Munich, Germany). Magnetic resonance angiogram/venogram (MAGNETOM Verio 3.0-T, Siemens AG, Munich, Germany) was performed to rule out PT caused by other (arteriovenous, arterial, and neoplastic) etiologies. The patient was free of ipsilateral transverse sinus stenosis, and both sides of the internal jugular vein outflow hemodynamics were obtained by neck ultrasound examination. The patient was free of intracranial hypertension and systemic diseases after lumbar puncture and blood work examinations. The dehiscence was defined as the absence of a bony plate overlying the sigmoid sinus vascular contour with at least three consecutive 0.6 mm axial CT cuts (Eisenman et al., 2018). The complete workflow of this study is illustrated in Figure 1.

### Intraoperative Setup of Condenser Microphone and Microphone Calibration

**FIGURE 2:**
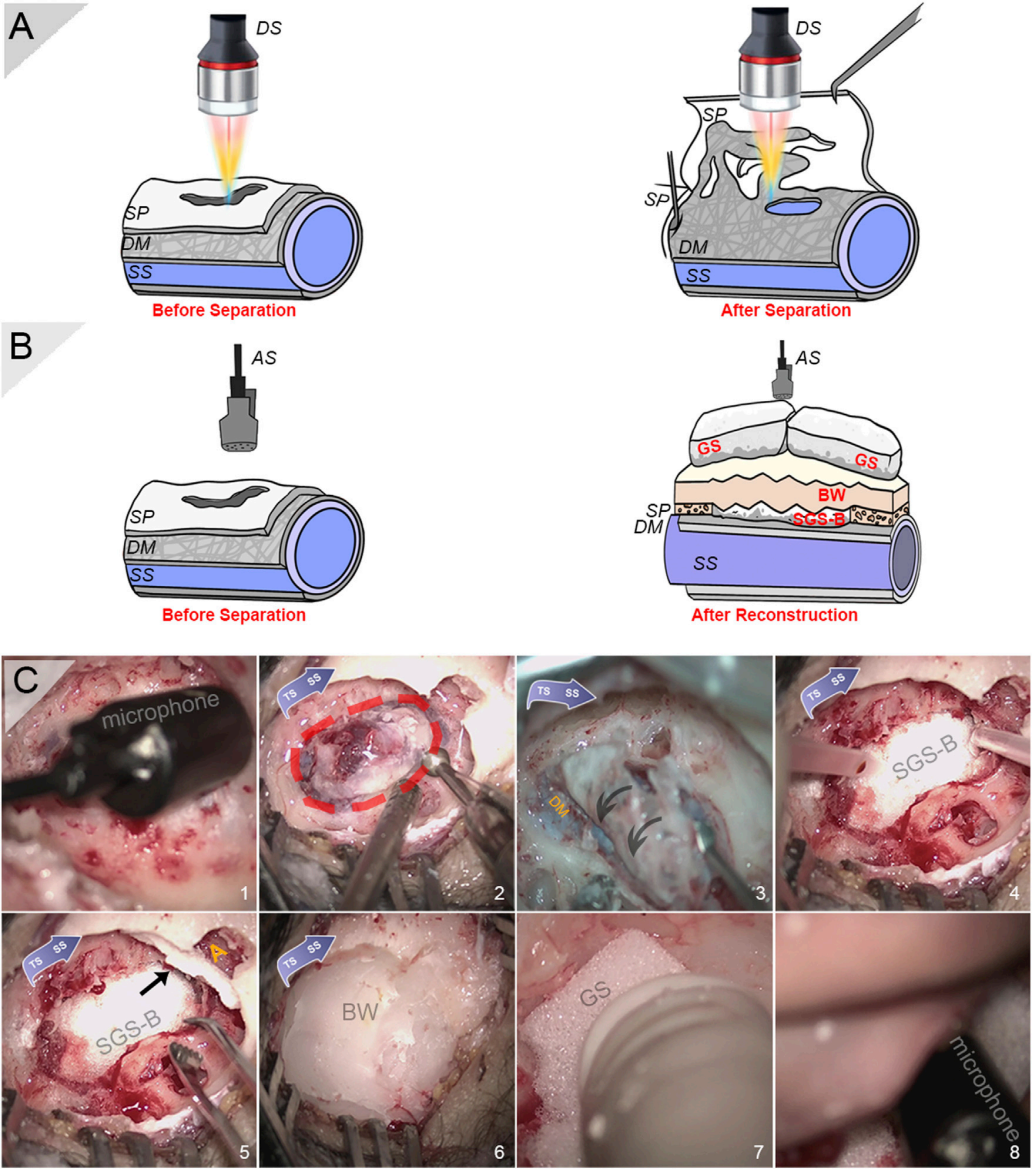
Intraoperative photographs and schematic diagrams for sensing applications. **(A)** Schematic diagram of intraoperative displacement sensing applications before and after separation of the vascular and osseous walls. **(B)** Schematic diagram of intraoperative acoustic sensing applications before and after reconstruction of sigmoid plate. **(C)** Intraoperative photographs of installation of condenser microphone, harvest of sigmoid plate specimen, and solidified gelatin sponge–bone wax repair technique (in sequence). (1) Intraoperative insertion of a condenser microphone after the exposure of sigmoid plate dehiscence to record PT. Note that the actual recording took place after the auricle and periosteal skin flap was reapproximated. (2) Harvest of the dehiscent bony plate. Note that a rectangular region was skeletonized to relieve the tightly fixated specimen. **(C)** Removal of the dehiscent sigmoid plate. Note that the dura mater fixates the vascular wall tightly to the concave surface of the sigmoid plate. **(D)** A squished solidified gelatin sponge-brick fixated using surgical glue to cover the transverse–sigmoid junction. **(E)** Another squished piece of solidified gelatin sponge-brick blocking the opening of the antrum (yellow A) alongside pieces of solidified gelatin sponge filling the air cells. **(F)** A thick layer of bone wax applied to cover and seal dead spaces inside the mastoid cavity. **(G)** The porous, soft gelatin sponge filling the remnant dead space. **(E)** Monitoring whether PT persists after multilayer resurfacing and quantification of surgical efficacy before closing the incision. DS indicates displacement sensor, SP indicates sigmoid plate, DM indicates dura mater, SS indicates sigmoid sinus, AS indicates acoustic sensor, SGS-B indicates solidified gelatin sponge-brick, GS indicates gelatin sponge, and BW indicates bone wax.

An omnidirectional condenser microphone ZJ005 MR (Zhijia Electronic Technology Co., Ltd., Dongguan, China) with a sensitivity of −34 dB and response frequency range of 50 Hz to 20 kHz was installed intraoperatively to obtain the *in vivo* acoustic characteristics of PT (Figure 2). The microphone sensor was disinfected using low-temperature plasma sterilization. As the dehiscence area was estimated after proper skeletonization without further obliterating the sigmoid plate, the microphone was inserted adjacent to the dehiscent area. The preoperative recording of PT was performed after the anteriorly lifted pedicle periosteal skin flap was reapproximated to reproduce the airtight mastoid cavity environment. After the reconstruction of the sigmoid plate, the microphone sensor was reinstalled for acoustic recording. The sonification of the captured audio data was analogous to that used in our previous research (Hsieh Y.-L. et al., 2021). The root mean square (RMS) amplitude and data conversion of decibel scales were achieved using the software MATLAB R2017a (MathWorks), according to the outcome of calibrating the microphone in an anechoic room.

### Intraoperative Setup of Confocal Laser Displacement Sensor

The intraoperative setup of the confocal laser displacement sensor has been described in detail previously (Hsieh and Wang, 2021). In brief, a confocal multicolor laser displacement sensor system CL-P030N (Keyence, Japan) with 0.25 μm resolution and high-precision linear measurement range ±0.72 μm was intraoperatively installed to measure the displacement of the dehiscent sigmoid plate after separation from the dura mater (Figure 2). The laser dot was centered, and the tested range was controlled at approximately 30 mm above the testing object. The angulation and sterilization of the sensor were performed according to our previous methods. The sampling cycle was set to 1,000 μs. CL-NavigatorN 1.4.0.0 (Keyence, Japan) was the software used to obtain displacement data. Displacement data were post-analyzed using a short-time Fourier transform; variational mode decomposition (VMD), i.e., Eq. 1; and continuous wavelet transform (CWT), i.e., Eq. 2, using MATLAB R2017a:

$$\underset{\left\{u_{k}\right\},\left\{\omega_{k}\right\}}{\min}\{\underset{k}{\sum }\|\partial_{t}[\left(\delta \left(t\right)+\frac{j}{\pi t}\right)*u_{k}\left(t\right)]e_{2}^{-jw_{k}t2}\},$$ (1)

$$s.\;t.\;\Sigma_{k}u_{k}=f,$$

where $\;\left\{u_{k}\right\}:=\left\{u_{1},\ldots ,u_{K}\right\}$ and $\left\{\omega_{k}\right\}:=\left\{\omega_{1},\ldots ,\omega_{K}\right\}$ are shorthand notations for entire modes and their center frequencies and $\Sigma :=\Sigma_{k=1}^{k}$ is the aggregation over all nodes. VMD decomposes an input signal into discrete sub-signals, which enhances the resolution of data visualization.

The CWT is a mathematical method that enhances the nonstationary signals and identifies the echoes representing defects, which allows the translation and scale parameter of the wavelets to vary continuously:

Xw(a,b)= 1|a|12∫−∞∞x(t)ψ¯(t−ba)dt, (2)

where ψ(t) is a continuous function in the time and frequency domains. A detailed description of the parameters used for signal analyses has been reported previously (Hsieh Y. L. et al., 2021; Hsieh Y.-L. et al., 2021; Hsieh and Wang, 2021).

### Biomaterial Specimens for Acoustical and Mechanistic Assessments

**FIGURE 3:**
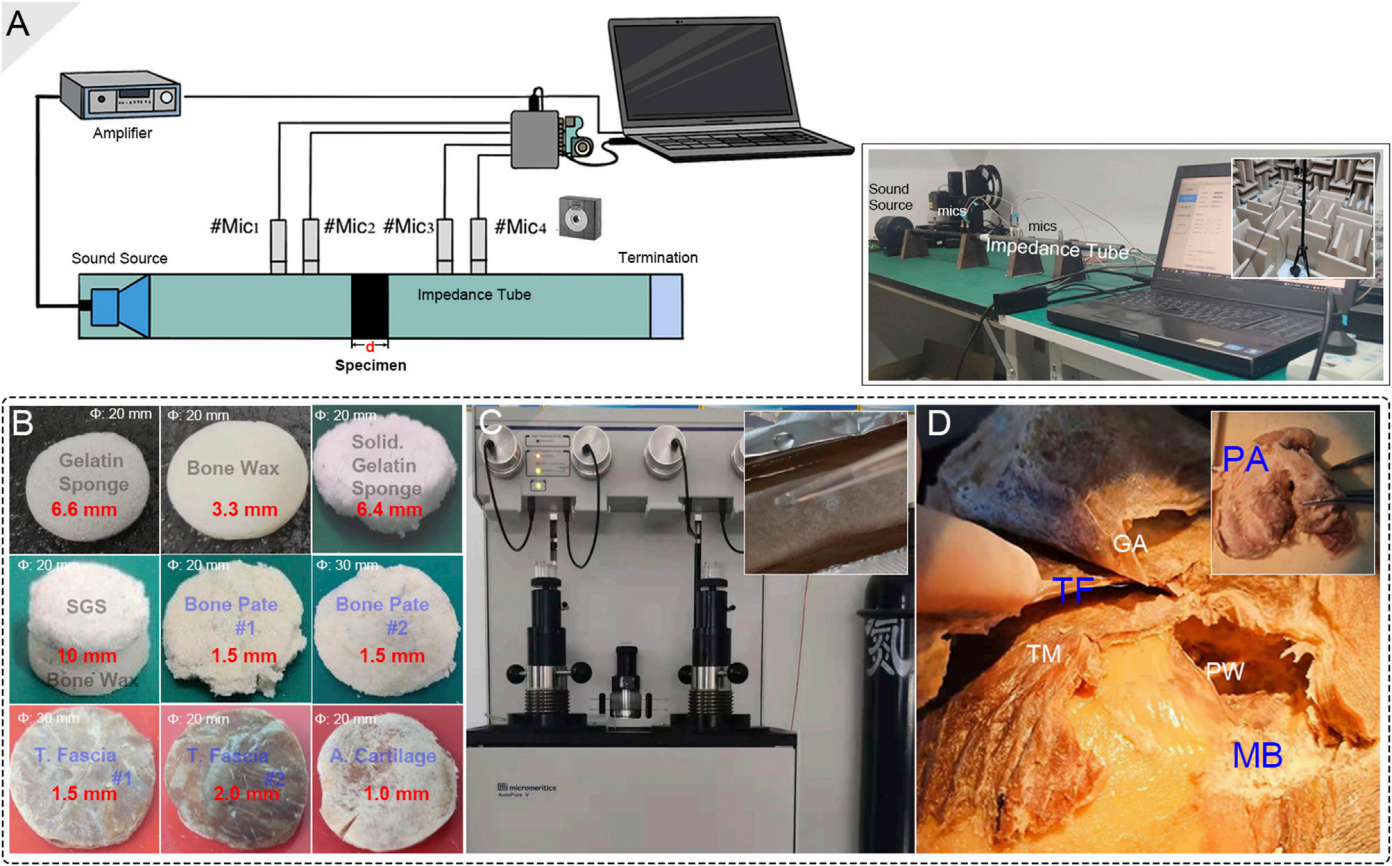
Biomaterial preparation and setup of the acoustical impedance investigation. **(A)** The schematic diagram of the impedance tube system setup. Notice that the deployed acoustic sensors were properly calibrated before each acoustical test using a calibrating device in an anechoic room. **(B)** The final testing biomaterials. The diameter and thickness of the materials are marked in white and red colors, respectively. **(C)** The mercury porosimeter and the making of a solidified-gelatin sponge. **(D)** The tested graft materials, i.e., bone pate, temporalis fascia, and auricular cartilage, were harvested from the cadaveric head. PA, TF, and MB indicate postauricular cartilage, temporalis fascia, and mastoid bone, respectively. GA, TM, and PW indicate galea aponeurotica, temporalis muscle, and posterior wall of the external auditory canal.

The tested biomaterials included (1) absorbable gelatin sponge (Jinling Pharmaceutical Company Ltd., Nanjing, China); (2) absorbable gelatin sponge smeared with surgical glue octylcyanoacrylate/*N*-butyl-cyanoacrylate (Stryker Corp., Kalamazoo, MI), which acts as a solidified gelatin sponge; (3) bone wax W810 (Ethicon LLC, Johnson & Johnson, NJ); (4) a compound of the solidified gelatin sponge and bone wax; (5) the glued bone pate; (6) temporalis fascia; and (7) postauricular cartilage. All specimens were cut into circular shapes with diameters of 20 or 30 mm for further impedance tube testing without altering their natural thickness. The temporalis fascia, postauricular cartilage, and bone pate were harvested from a fresh frozen cadaveric head, as shown in Figure 3. The natural thicknesses of these materials were reserved for all acoustic assessments.

### Determination of Sound Absorption and Impedance in Impedance Tube

The acoustic characteristics of the specimens were determined using an impedance tube system. The diameter of the aluminum alloy impedance tube Φ for biomaterial testing was 20 or 30 mm according to the diameter of the specimens. Four free-field microphones (Type 4939, Brüel & Kjær, Denmark) with a tested sensitivity of 10 mV/Pa were placed at the front and back of the specimen in two pairs. The planar wave sound source was generated using a speaker (JBL). The average transmission loss of sound impedance and the estimated density of the biomaterials were investigated using the transmission loss formula below:

$$TL=20\log_{10}\left\{\frac{1}{2}\left|A_{12}+\frac{B_{23}}{\rho c}+\rho c\cdot C_{23}+D_{23}\right|\right\}+10\log_{10}\left(\frac{S_{i}}{S_{o}}\right),$$ (3)

where *ρ* is the air density, *c* is the speed of sound, and *S*_*i*_ and *S*_*o*_ are the cross-sectional areas of the impedance tube inlet and outlet, respectively. The sound absorption factor α is defined by

$$\alpha =\frac{E_{\alpha }+E_{t}}{E}=\frac{E-E_{r}}{E}=1-r,$$ (4)

where E is the total sound energy, $E_{\alpha }$ is the absorbed energy, $E_{t}$ is the transmitted energy, $E_{r}$ is the reflected energy, and r is the reflection coefficient. To obtain the average sound absorption factor, the absorbance coefficients of the octave of six center frequencies 125 Hz, 250 Hz, 500 Hz, 1 kHz, 2 kHz, and 4 kHz were averaged. For the average transmission loss, the octave of 13 center frequencies 125 Hz, 160 Hz, 200 Hz, 250 Hz, 315 Hz, 400 Hz, 500 Hz, 630 Hz, 800 Hz, 1 kHz, 1.25 kHz, 1.6 kHz, and 2 kHz were averaged.

### μCT and Scanning Electron Microscope

The harvested bony plate was left in a fixative 4% paraformaldehyde solution for 24 h and then immersed in a solution of 75% alcohol for dehydration. A SkyScan 1176 micro-CT (μCT) system (Bruker micro-CT, Belgium) was used to scan the harvested bony plate. Version 1.6 of the NRecon software was used for 3D reconstruction and viewing of images. Mimics 19.0 and 3-Matic 11.0 (Materialise, Belgium) were used to analyze the wall thickness of the bony plate.

The gelatin sponge, solidified gelatin sponge, bone wax, and harvested dehiscent sigmoid plate were observed using a Regulus 8100 ultra-high-resolution field-emission scanning electron microscope (SEM) (HITACHI, Japan) at 3.0 kV.

### Mercury Porosimeter

The pore size distribution was determined using an AutoPore V mercury porosimeter (Micromeritics Instrument Corporation, Norcross, GA, United States) based on

$$P=\pi r^{2}p=P_{s}-2\pi r\sigma \cos\theta \pi r^{2}p=-2\pi r\sigma \cos\theta ,$$ (5)

where *θ* represents the contact angle of mercury and *r* indicates the surface tension of mercury. The contact angle was maintained at >90°. The largest pressure *P* was 400 MPa, and the range of detection was 0.003–950 μm. When *P* > *P*_*s*_, Washburn’s equation is established:

$$P_{capillary}=\frac{-2\sigma \cdot cos\theta }{r}$$. (6)

The intrusion volume, pore area, bulk density, porosity of the soft/solidified gelatin sponges and bone wax were investigated.

### Finite Element Models, Doppler Ultrasonography, and Coupled Computational Simulations for Operative Biomaterial Applications

**FIGURE 4:**
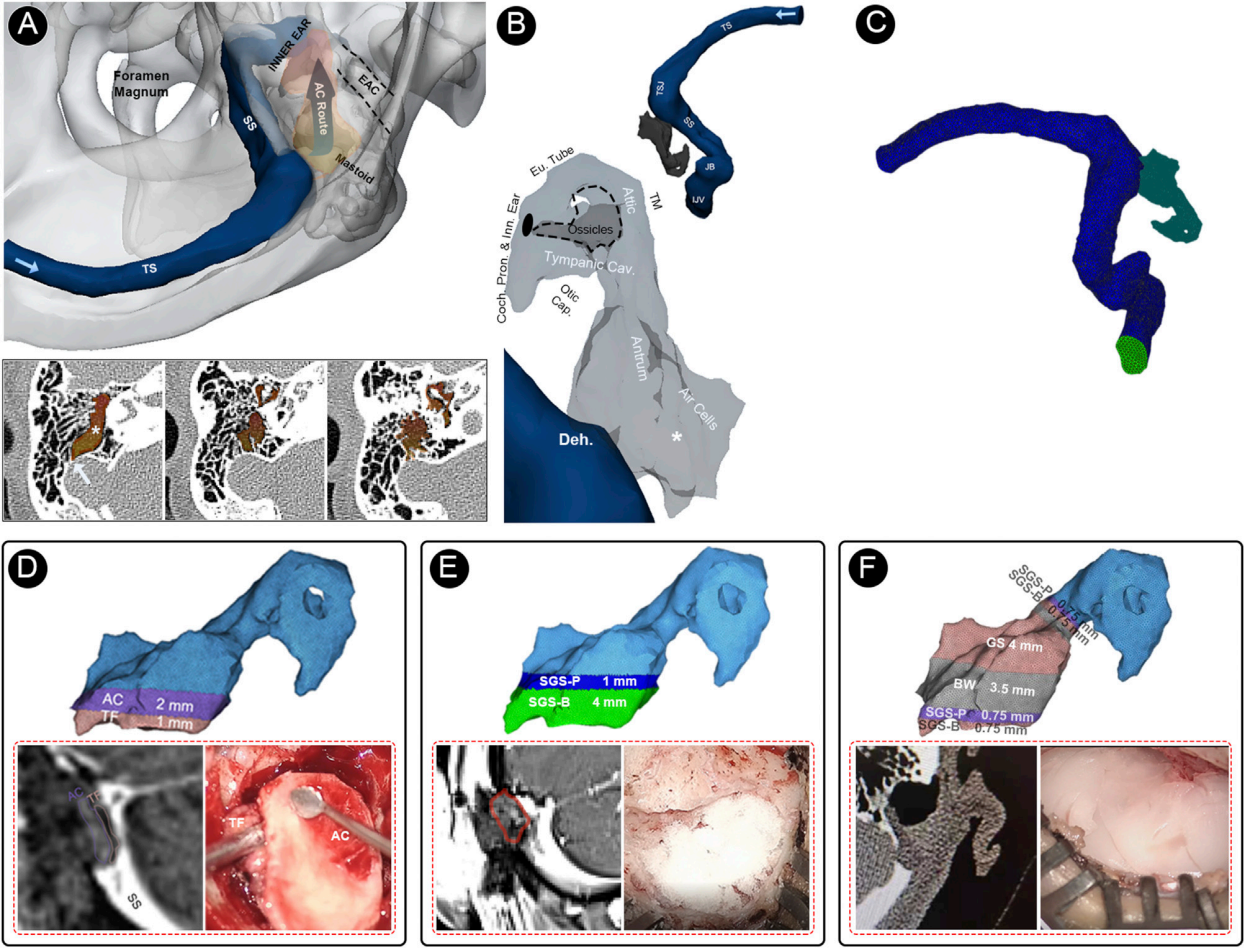
Multi-sensing platforms and computational setup of this study. **(A)** Multi-sensing platforms to examine displacement and acoustic characteristics of vascular wall vibration and airborne sound of the blood flow motion. **(B)** Relative anatomical structures of the air-conduction transmission route of PT caused by sigmoid sinus wall dehiscence. **(C)** Finite element models of the flow acoustic fields. **(D)** The finite element model, representative radiologic imaging, and representative intraoperative photograph of the temporalis fascia–auricular cartilage (TF-AC) surgical repair method. **(E)** The finite element model, representative radiologic imaging, and representative intraoperative photograph of the solidified gelatin sponge-brick (SGS-B) and solidified gelatin sponge-pieces (SGS-P) method. **(F)** The finite element model, representative radiologic imaging, and representative intraoperative photograph of the solidified gelatin sponge-bone wax (SGS-BW) surgical repair method.

To investigate the efficacy of sound insulation in different biomaterial applications, the CFD technique proposed above (see Figure 4 for modeling and details of biomaterial applications) was used to investigate the currently implemented solidified gelatin sponge–bone wax technique and two previous surgical methods used by our research group, i.e., the auricular cartilage–temporalis fascia technique and gelatin sponge complex technique. The simulated flow sound source was applied to all three reconstruction techniques to juxtapose the efficacy of transtemporal sound insulation.

The patient-specific finite element models were reconstructed using Mimics 19.0 and 3-Matic 11.0 (Materialise, Belgium). The ipsilateral transverse-sigmoid sinus and the aerial space inside the mastoid cavity were reconstructed. The branches of the ipsilateral transverse-sigmoid sinus and the aerial spaces that were irrelevant to the direction of PT transmission were removed. The grids were meshed using ANSA v22.0.1. A total of 1,921,703 and 156,526 cells were constructed for the flow and acoustic domains, respectively. Eight layers with a boundary layer thickness 0.02 mm were reconstructed. Star-CCM+ 2020 (Siemens, Germany) was used to perform the flow field, acoustic field, and fluid–structure interaction analyses. In the flow field simulation, we adopted the transient laminar method to calculate the Navier–Stokes equations (Eqs 7, 8):

∇⋅u=0 (7)

$$\rho \frac{\partial u}{\partial \text{t}}+\rho u\cdot \nabla u=-\nabla p+\nabla \cdot \tau +\rho g,$$ (8)

where u is the 3D velocity vector of the incompressible Newtonian flow fluid blood, the density of blood *ρ* was 1,050 kg/m^3^, and the dynamic viscosity μ was 0.00345 Pa s. The estimated Reynolds number in this case was 1,065. The inlet flow velocity was set according to the patient’s cervical Doppler results (MyLab ClassC, Esaote SpA, Genoa, Italy). The transient time–velocity data were acquired using Doppler ultrasound and transformed for the setup of the velocity inlet. The time steps were set at 0.00005 s. Two pulsatory cycles of 1.46 s were calculated, with the second pulsatory cycle established as the representative cycle.

After the solution of the flow domain was acquired, the Actran 2020 (MSC, Free Field Technology, Belgium) was used to calculate the vibroacoustic and hydroacoustic sound sources and transmittance inside the mastoid acoustic domains. The boundary conditions (solid structure density, Young’s modulus, and Poisson’s ratio) of biomaterials and vascular structures adopted for coupled CFD simulation were established based on impedance tube test results and previous literature (Table 1) (Lakes, 1987; Qi et al., 2006; Wellisz et al., 2008; Curtis et al., 2011; Kuo et al., 2016; Long et al., 2017; Zwirner et al., 2021). According to the thickness of the transverse-sigmoid sinus junction of the cadaveric specimen, the moving sigmoid sinus vascular wall was set to 0.3 mm, wherein Young’s modulus was 1.26 MPa, Poisson’s ratio was 0.3, and the solid structure density was 1,050 kg/m^3^ (Tian et al., 2017). The outlet pressure was set to zero. The Lighthill sound analogy theory was used to simulate the hydroacoustic field (Lighthill, 1952; Howe, 2003) (Eq. 9):

$$\frac{\partial \rho }{\partial t^{2}}-c_{0}^{2}\nabla^{2}\rho =\frac{\partial^{2}T_{ij}}{\partial y_{i}\partial y_{j}},$$ (9)

where $T_{ij}$ is the Lighthill tensor (Eq. 10):

$$T_{ij}=\rho u_{i}u_{j}+p\delta_{ij}+\tau_{ij}-c_{0}^{2}\nabla^{2}\rho \delta_{ij}.$$ (10)

**TABLE 1:** The acoustical and mechanical properties of grafts and biomaterials.

	Gelatin sponge	Solidified gelatin sponge	Bone wax	Bone pate	Temporalis fascia	Auricular cartilage
Specimen Thickness (mm)	6.6	6.4	3.3	1.5	1.5/2.0	1.0
Diameter (mm)	20	20	20	20/30	20/30	20
Density (kg/m^3^)	34.2^b^	368.9^b^	945.3^b^	420/465^c^	703/536^c^	1,130^c^
^a^ $\bar{\alpha }$ /average TL (dB)	0.171	10.9	45.3	7.0/3.6	9.7/18.5	15.2
Young’s Modulus (MPa)	0.170	0.0599	0.146	12,000	36	1.66
Poisson’s Ratio	0.3	0.3	0.37	0.3	0.3	0.47

Note that two bone pate and temporalis fascia specimens were tested due to high heterogeneity, and 703 kg/m^3^ was elected as the density of temporalis fascia adopted for CFD, simulation.

a $\bar{\alpha }$ indicates average absorbance factor (gelatin sponge) and TL indicates volume of transmission loss at the specific specimen thickness.

b Values acquired from porosimeter outcomes.

c Values estimated by impedance tube testing.

The Lighthill acoustic analogy was carried out by solving the Lighthill equation using an integral solution to the inhomogeneous wave equation (Eq. 11) demonstrated below (Howe, 2003):

$$4\pi c_{0}^{2}\rho ’x,\;t=\frac{\partial^{2}}{\partial x_{i}\partial x_{j}}\int \int \int_{v}\left[\frac{T_{ij}}{\left|x-y\right|}\right],$$ (11)

where *V* indicates acoustic radiation. The transformation of the pressure fluctuation and flow velocity was simulated using a quadrupole sound source. The density and acoustic velocity of air were 1.139 kg/m^3^ and 340 m/s, respectively. A virtual microphone was set at the inner ear region inside the tympanum to collect data on flow-borne and fluid–structure interaction sounds. The obtained vibroacoustic and hydroacoustic sounds were post-processed using A-weighting. The reference pressure was set to 20 μPa.

### Statistical Analysis

Statistical analysis was performed using RStudio software (RStudio, Boston, MA, United States). After the assessment of data normality using the Shapiro–Wilk test, the differences between the continuous data were compared using the Mann–Whitney *U*-test. Five consecutive seconds of displacement and acoustic data were compared. The significance level was set at *p* < 0.05.

## Results

### Morphological Characteristics of Dehiscent Sigmoid Plate

**FIGURE 5:**
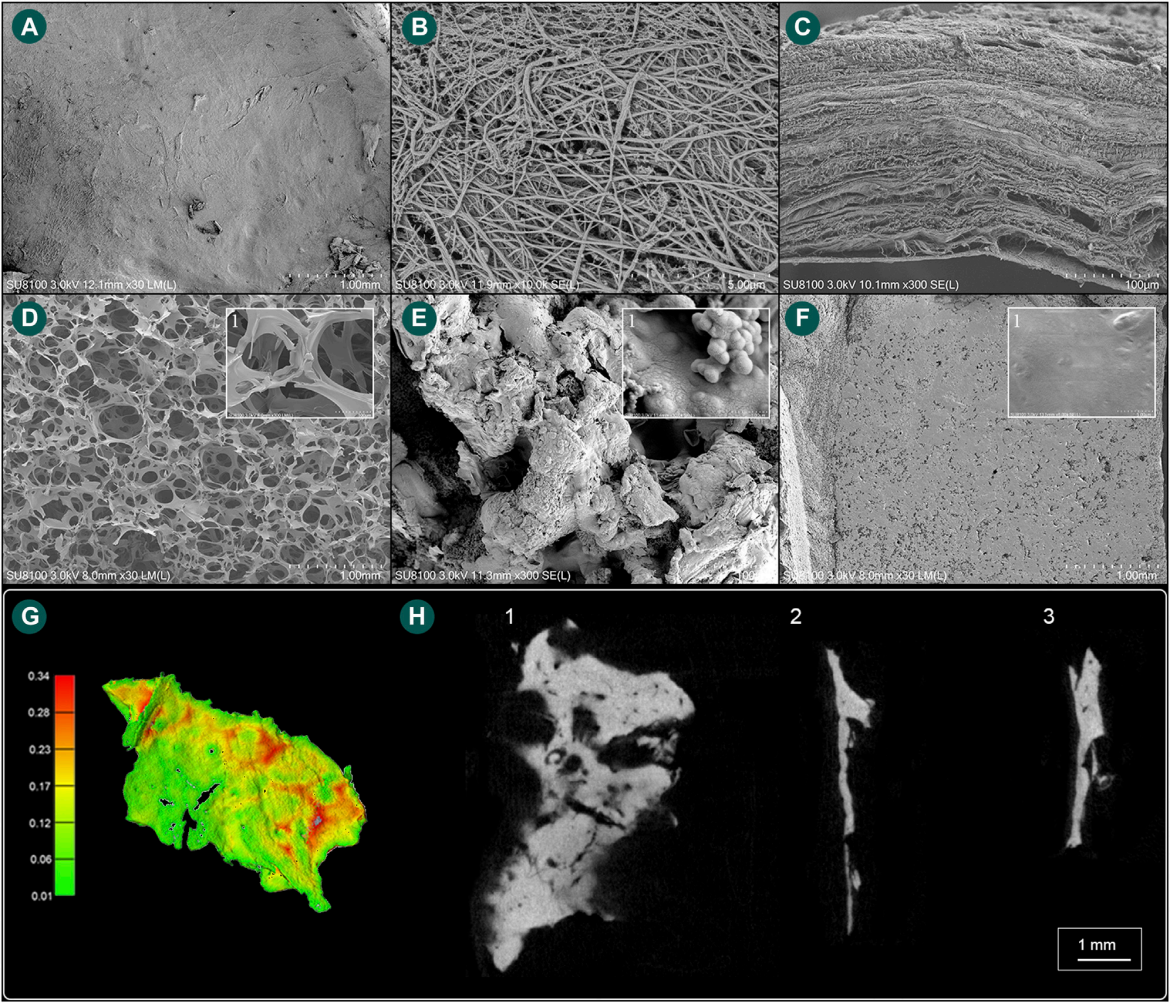
Scanning electron microscope and microstructural characteristics of the harvested sigmoid plate and biomaterial specimens. **(A)** The concave surface of the harvested sigmoid plate (magnification ×30). **(B)** Layers of dura mater (magnification ×10,000). **(C)** Lateral view of the harvested transverse–sigmoid junction (magnification ×300). **(D)** Gross view (magnification ×30) of the soft gelatin sponge and (1) microscopic view (magnification ×300) of pore structures of the soft gelatin sponge. **(E)** Gross view (magnification ×300) of the solidified gelatin sponge and (1) microscopic view (magnification ×30,000) of pore structures of the solidified gelatin sponge. **(F)** Gross view (magnification ×30) of the bone wax and (1) microscopic view (magnification ×6,000) of the surface of the bone wax. **(G)** Computed wall thickness of the concave surface of the harvested sigmoid plate specimen. **(H)** (1) μCT horizontal view of the harvested sigmoid plate. (2) μCT lateral view of the harvested sigmoid plate. (3) μCT topical view of the harvested sigmoid plate.

The area of the harvested sigmoid plate was approximately 5.8 × 3.8 mm^2^, and the surface area was 54.02 mm^2^. The median wall thickness was 0.12 (0.068/0.18) mm, and the largest measured thickness was 0.92 mm. Under the SEM examination, layers of dura mater were observed attached to the concave surface of the harvested sigmoid plate. Microscopic morphological characteristics are shown in Figure 5.

### Displacement Characteristics of Dehiscent Sigmoid Plate

**FIGURE 6:**
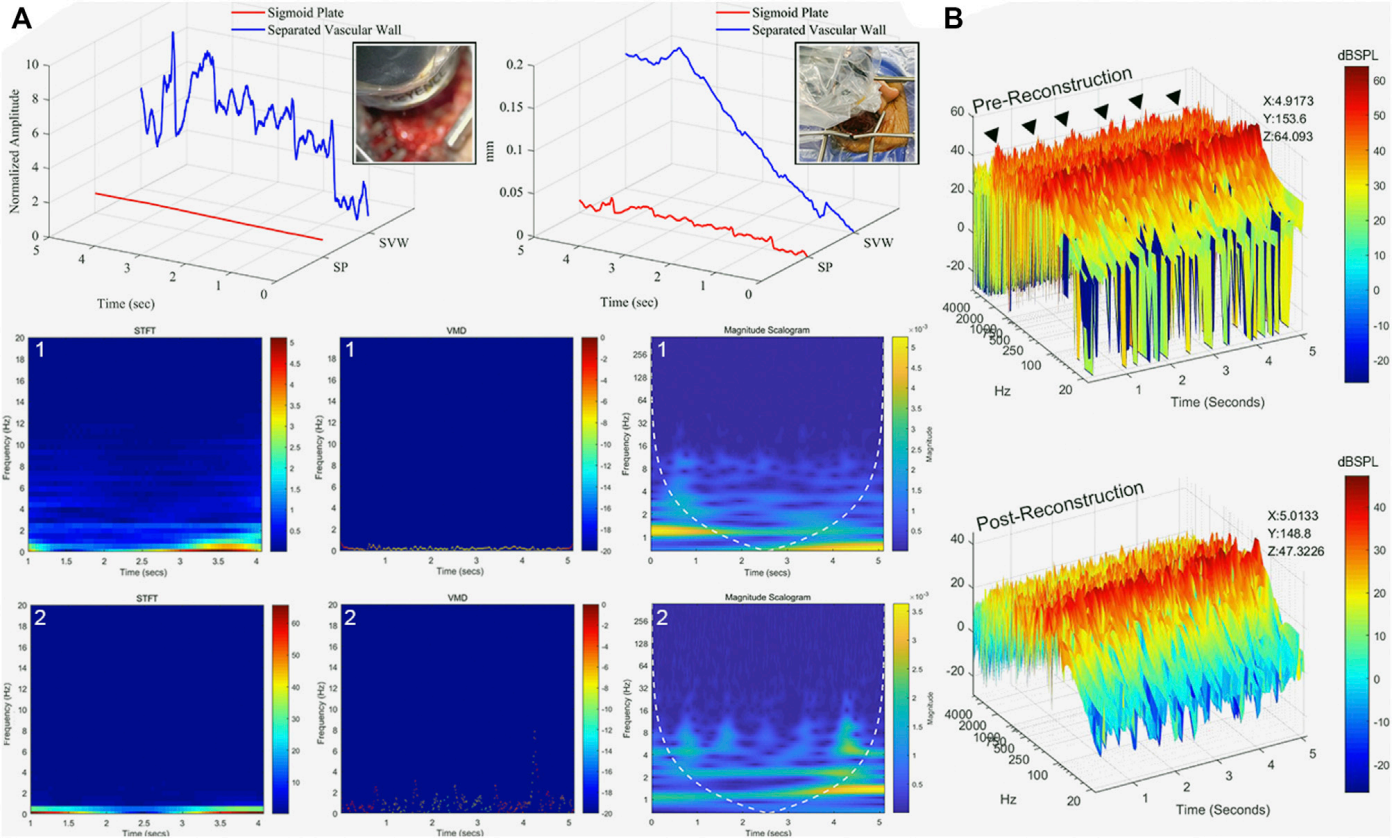
Displacement and acoustic characteristics of sigmoid plate and PT. **(A)** Displacement characteristics of sigmoid plate and vascular wall after osseous and vascular wall separation in five continuous seconds. (1) The displacement measured at the sigmoid plate before separation from the vascular wall. The displacement data presented in short-time Fourier transform (STFT), VMD, and the outcome of CWT (magnitude scalogram). (2) The displacement measured at the surface of the sigmoid sinus vascular wall. Observed pulse-synchronous signals marked with black triangles. The displacement data presented in STFT, VMD, and CWT. **(B)** Recorded acoustic signal of pre- and post-reconstruction of the sigmoid plate. The peak amplitude (z-axis, dB SPL) at specific time (x-axis) and frequency (y-axis) is marked.

Acoustic and vibroacoustic sensing outcomes are shown in Figure 6. Before the removal of the dehiscent sigmoid plate overlying the sigmoid sinus, the median displacement of the sigmoid plate was 0.012 (0.0087/0.014) mm, whereas the median displacement of the sigmoid sinus vascular wall after separation from the sigmoid plate was 0.092 (0.039/0.15) mm. There was a significant difference between the displacement before and after separation of the osseous and vascular walls (Mann–Whitney *U*-test, *p* < 0.01). The transformation of vibration signals using short-time Fourier transform, VMD, and CWT techniques is shown in Figure 6, where the major frequency of the detected displacement was predominantly below an audible range.

### Mechanical Properties and Acoustical Characteristics of Grafts and Biomaterials

**FIGURE 7:**
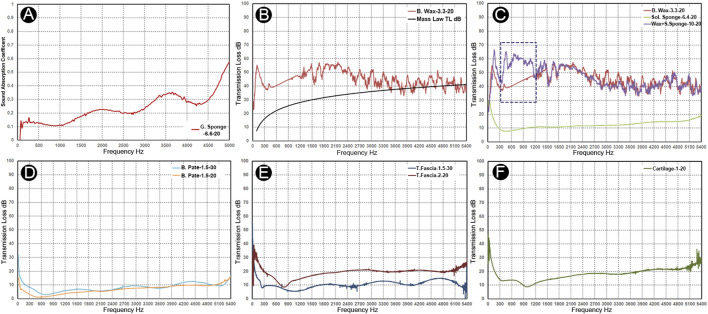
Acoustic absorption and insulation properties of the grafts and biomaterials. Nomenclature: materials-thickness-diameter. **(A)** Absorbent coefficient vs. frequency graph of soft gelatin sponge. **(B)** Transmission loss vs. frequency graph of bone wax. **(C)** Transmission loss of bone wax (red), solidified gelatin sponge (yellow), and solidified gelatin sponge–bone wax complex (purple). Notice an evident increase of the sound insulation volume at 200–1,200 Hz of the complex material specimen compared to the bone wax specimen (blue dotted rectangle). **(D)** Transmission loss vs. frequency graph of the bone pate samples. **(E)** Transmission loss vs. frequency graph of the two temporalis fascia specimens. **(F)** Transmission loss vs. frequency graph of the auricular cartilage.

The mechanical properties and acoustic characteristics of the specimens are shown in Table 1 and Figure 7. The total intrusion volume of the porous gelatin sponge was 28.28 ml/g, and the total pore area was 0.773 m^2^/g. The tested sample density was 34.2 kg/m^3^. The porosity and interstitial porosity were 96.7% and 47.6%, respectively. The absorptance coefficient increased as the frequency of the given stimuli increased, and the average sound absorption factor was 0.171. After application of the surgical glue, the bulk density of the solidified gelatin sponge was altered to 368.9 kg/m^3^. The average pore diameter (4V/A) decreased from 0.146 to 0.0036 mm. The porosity decreased to 67.4%, and the average sound insulation volume of the solidified sponge specimen was 10.9 dB. For the bone wax, the total intrusion volume was 0.0376 ml/g, and the total pore area was 0.190 m^2^/g. The average pore diameter (4V/A) was 792.5 nm. The tested bulk density of bone wax was 945.3 kg/m^3^. The porosity and interstitial porosity were 3.5% and 25.9%, respectively. The average sound insulation volume of the bone wax specimen was 45.3 dB. By conjoining the solidified gelatin sponge with bone wax, the sound insulation volume increased greatly at 200–1,200 Hz, possibly due to impedance mismatch and the formation of the interstitial air layer. The average sound insulation volume of the solidified gelatin sponge–bone wax compound specimen was 54.7 dB. There was also an evident valley value in the low-frequency range caused by the stiffness effect, which is associated with the inflexibility of the bone wax.

In human specimens harvested from the cadaveric head, the estimated densities of the two glued bone pate specimens of different thicknesses were 420 and 465 kg/m^3^, and the average sound insulation volumes were 7.0 and 3.6 dB. Because the density of bone pate samples varied, there was a volatility in the insulation volume between the two samples. Unlike the bone pate, the transmission loss in the temporalis fascia samples was proportional to the increase in frequency. The estimated densities of the temporalis fascia with 1.5 and 2.0 mm thickness were 703 and 536 kg/m^3^, respectively. As there were stiffness and thickness differences between samples, the transmission loss in the low-frequency range <900 Hz was more salient. The average transmission loss was 9.7 and 18.5 dB in the 1.5 and 2.0 mm temporalis fascia, respectively. Similar to the temporalis fascia, the transmission loss in the cartilage was proportional to the increase in frequency. The estimated density of the auricular cartilage specimen was 1,130 kg/m^3^, and the average transmission loss was 15.2 dB. The transmission loss was larger at frequencies below 300 Hz, and due to its certain flexibility, the first-order resonance at 900–1,200 Hz was observed, resulting in a decrease in the transmission loss at the corresponding frequency range.

### Results of Intraoperative Microphone Detection

See Figure 6 and Supplementary materials for the results and intraoperative recordings. The pre-reconstruction RMS amplitude was 0.0032 (see also Supplementary Audio S1), whereas the post-reconstruction RMS amplitude was 0.00039 (see also Supplementary Material S2). There was a significant difference in pre- and post-reconstruction amplitudes (Mann–Whitney *U*-test, *p* < 0.01). The intraoperative measured pre-reconstruction peak amplitude was 64.09 dB SPL, whereas the post-reconstruction peak amplitude was 47.3 dB SPL. The frequency of the PT undulates below 2 kHz. PT was completely resolved by a drop of 16.7 dB in the peak PT amplitude.

### Results of Coupled CFD Simulation in Different Transtemporal Repair Techniques

**FIGURE 8:**
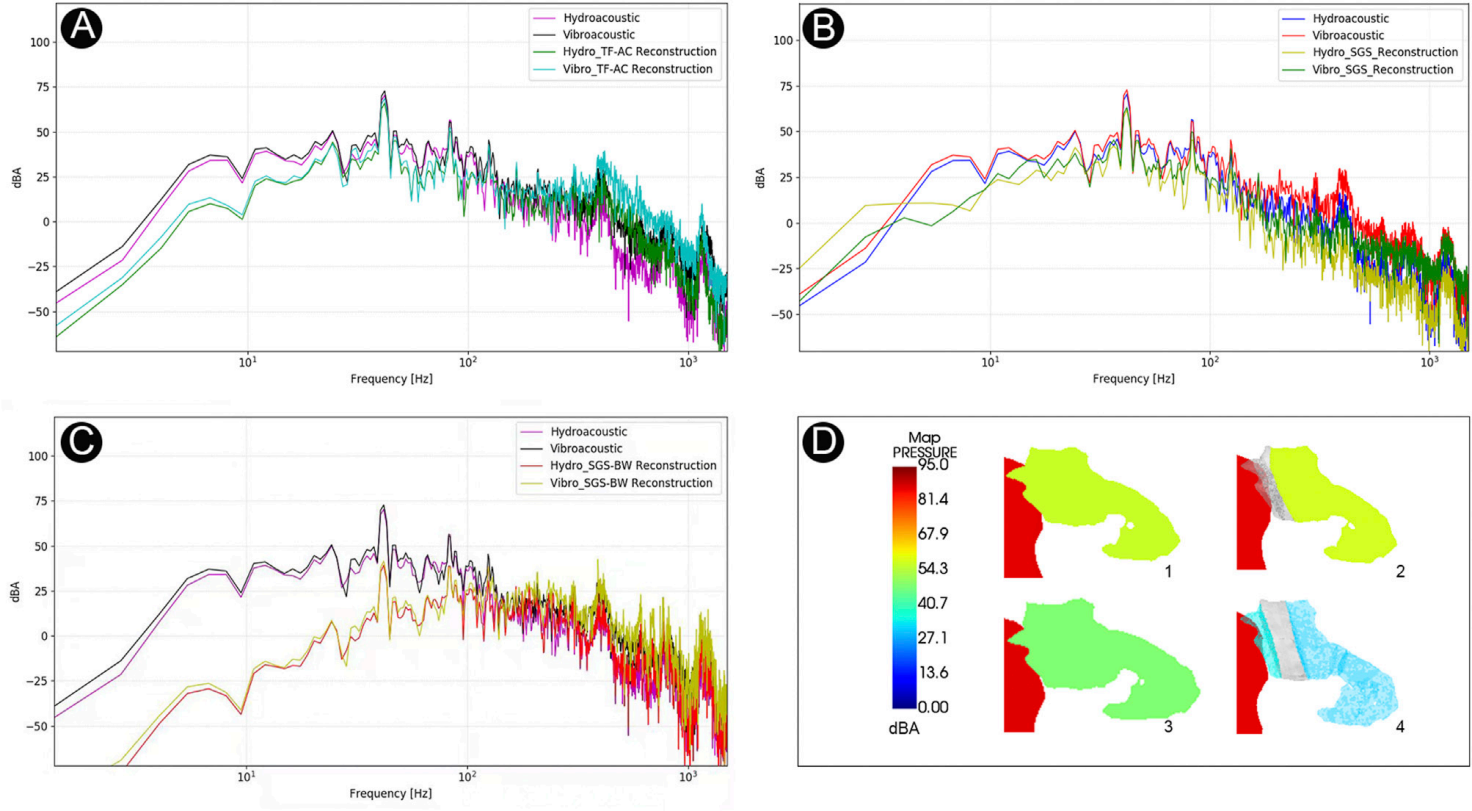
Computational results of amplitude of PT and sound insulation results among different transtemporal repair techniques. **(A)** The amplitude spectrum of surgical efficacy after temporalis fascia–auricular cartilage (TF-AC) surgical material repair. **(B)** The amplitude spectrum of surgical efficacy after solidified gelatin sponge (SGS) surgical material repair. **(C)** The amplitude spectrum of surgical efficacy after solidified gelatin sponge–bone wax (SGS-BW) surgical material repair. **(D)** Visualization of PT transmission inside the middle-ear cavity.

The data of coupled CFD simulations are shown in Figure 8. The peak amplitude of generated vibroacoustic and hydroacoustic sounds measured before sigmoid plate reconstruction was 72.8 and 70.4 dB at 42.0 Hz, respectively. After sigmoid plate reconstruction using the temporalis fascia–auricular cartilage grafts, the peak amplitude of vibroacoustic and hydroacoustic sounds was 68.6 and 65.9 dB, and the range of vibroacoustic and hydroacoustic PT amplitude reduction was 4.2 and 4.5 dB, respectively. Compared to the autologous grafts, the solidified gelatin sponge brick and pieces resulted in a 9.7 and 8.9 dB reduction in vibroacoustic and hydroacoustic amplitudes, with the largest amplitude being 63.1 and 61.5 dB, respectively. The most effective surgical repair material was the solidified gelatin sponge–bone wax compound, which reduced the peak amplitude of the generated vibroacoustic and hydroacoustic sounds by 30.4 and 31.2 dB, respectively. The peak amplitude of generated vibroacoustic and hydroacoustic sounds was 42.4 and 39.2 dB after reconstruction using composite biomaterials.

## Discussion

### Vibroacoustic and Hydroacoustic Sources of PT

This study is the first to integrate *in vivo* detection of PT with *in vitro* and computational therapeutic efficacy of biomaterials and their ability to preclude vibroacoustic and hydroacoustic sounds using multi-sensing platforms. According to previous coupled CFD studies focusing on vibroacoustic sound generation, Tian et al. and Mu et al. found that the sigmoid sinus wall vibration produced 56.9 and 75.6 dB at 681 and 1,200 Hz, respectively. They suggested that the flow pulsation exerts pressure on the vascular wall, generating vibroacoustic waves. Despite the existence of a stark difference in frequency between the current and other CFD studies, the frequency of *in vivo* captured PT signals and psychoacoustically matched results suggests that the major power of PT fluctuates in the low-frequency range. Nevertheless, the possibility that PT arises because of the fluid–structure interaction of the entire sigmoid plate remains unknown. Further sensing applications to ascertain the source of the PT are warranted.

According to the current *in vivo* displacement sensing results, the major frequency of flow-induced vascular displacement was below the audible frequency range. This outcome contradicts the current coupled CFD outcomes and a few *in vitro*/computational studies (Tian et al., 2019; Mu et al., 2021). There are two plausible reasons for this divergence: first, the sigmoid sinus vascular wall is tightly fixated onto the concave surface of the sigmoid plate connected by the dura mater situated in between, which can be difficult to reproduce using experimental and computational methods. Tissues overlying the sigmoid sinus wall may directly influence the movement of the vascular wall. The second possibility is overestimation of the dehiscent area of the sigmoid plate. Despite the fact that dehiscence is determined using traditional CT scans, a bone thickness of less than 0.6 mm overlying the sigmoid sinus can be falsely interpreted as dehiscence. In the current study, the thinned bone tissue overlying the outer surface of the sigmoid sinus remains mostly intact, where the median wall thickness of the harvested sigmoid plate was 0.12 mm according to the more precise μCT analysis. The major frequency of vascular wall displacement fell below 20 Hz, even after the separation of the osseous and vascular walls. Together, these findings and indications suggest that the displacement of the sigmoid sinus vascular wall may be a less predominant factor causing PT and that the sound wave energy permeating through the weakened bone is likely a more dominant causative factor for PT.

### Therapeutic Effect and Clinical Implications for Transtemporal Reconstruction Methods

Transtemporal reconstruction surgery targets the reduction of vibration of the sigmoid sinus wall and reconstruction of the soundproof sigmoid plate. Eisenman and Song’s research teams relied on the combination of autologous grafts and synthetic bone cement to resurface the defective bony plate, both of which have high surgical success rates (Eisenman et al., 2018; Lee et al., 2020). In our previous clinical findings, merely using a rigid cartilage to fortify the defective sigmoid plate without altering the intrasinus hemodynamics failed to eliminate PT (Hsieh and Wang, 2020; Hsieh Y. L. et al., 2021). This is verified by the current finding that the cartilage–temporalis fascia method reduces the vibroacoustic and hydroacoustic amplitudes by 4.5 and 4.2 dB, which may be inadequate to eliminate PT considering a mean 17.7 dB of PT loudness above the hearing threshold measured on 55 subjects (Hsieh Y.-L. et al., 2021). The current multilayer reconstructive solidified gelatin sponge–bone wax technique resulted in a reduction of over 30 dB in the PT amplitude. Since PT is transmitted by air, establishing solid sound insulation materials such as bone wax at the aditus not only provides a secondary perimeter to preclude the PT transmission but also prevents material migration. Nevertheless, in some circumstances, resurfacing dehiscence without addressing flow anomalies may fail to eliminate PT (Lee et al., 2020; Hsieh Y. L. et al., 2021). The adoption of an appropriate surgical method should be assessed meticulously preoperatively and executed properly by otologists intraoperatively to ensure the therapeutic efficacy of transtemporal methods.

The current intraoperative condenser microphone showed that PT was transmitted by air, which was consistent with previous *trans*-ear canal recording methods described by other authors (Kim et al., 2016). The 16.7 dB reduction in peak PT amplitude measured after reconstruction of the sigmoid plate in this study was close to a median of 20.8 dB in six operative subjects measured by Song’s research team (Lee et al., 2020). These sensing applications indicate that the aerial transmission of PT plays a significant role in the patient’s perception of the vascular sound, which is highly indicative of transtemporal reconstruction surgery.

### Grafts and Biomaterials for the Sound Insulation of PT: Otologist’s Perspective of Adopting Passive Noise Reduction

Although there are omnifarious choices of repair materials with higher density, otologists should consider that applying materials with higher areal density reduces the aerodynamic sound permeance. Sound permeation complies with the mass law. The temporalis fascia and bone chip are the most commonly used grafts for sigmoid sinus resurfacing (Wang et al., 2017). Nevertheless, maintaining a throughout flatness and fortifying the sigmoid plate with higher planar thickness using postauricular cartilage and temporalis fascia can be arduous during surgery. This explains why multilayers of bone wax and solidified gelatin sponge outperform a thin piece of fascia and cartilage in enhancing the transmission loss of PT. In addition, harvesting autologous materials such as the temporalis fascia and postauricular cartilage may induce postoperative pain or numbness due to additional incisions (Guo and Wang, 2015). Adding external biocompatible materials helps to reduce operative invasiveness and achieve the desired therapeutic effect concurrently.

Bone wax and bone cement are biocompatible biomaterials that possess a certain degree of stiffness. They are easy to manipulate or reshape and are highly customizable to create a soundproofing barrier with arbitrary thickness. Therefore, compared to autologous materials, the use of external biomaterials can be beneficial when running out of autologous materials. However, according to our previous clinical experience, bone wax and applied materials can migrate if they are not glued properly or rigorously fixated to a hard surface (Bhatnagar et al., 2020). Therefore, it is necessary to fixate bone wax properly. Considering that the harmonic range of PT can extend over 1.5 kHz in some cases (Hsieh Y.-L. et al., 2021), porous materials can play a role in absorbing a higher frequency range of PT. The combined use of soft and hardened gelatin sponge composition has proven highly effective in previous surgical series. Although the bulk density of soft gelatin sponge cannot reach as high as bone wax or bone cement, Gelfoam is a highly shapable stuffing material that is convenient to fixate with autologous grafts, fill dead spaces, and solidify to boost the areal density of repairing materials whenever the surgical gel is administered. The hardened gelatin sponge, having an areal density close to that of the autologous bone pate, can also replace the bone pate when the collection of bone pate is inadequate.

### Limitations and Further Study Directions

This study has some limitations. In multi-sensing investigations, the distance between the condenser microphone and the vascular wall was unmeasurable because of the need to reapproximate the pedicle skin flap. Although a specific measurement method for the confocal laser displacement sensor had been developed previously, some measurement biases may be present in gauging the vascular wall displacements. Additionally, tissues derived from the cadaveric head may not perfectly represent those of the *in vivo* state. In general, the autologous grafts had non-uniform thickness, rendering the estimation of density difficult. It is noteworthy that the heterogeneity of grafts harvested from different locations among subjects can also vary greatly, which otologists should be aware of. Analogously, biosynthetic biomaterials may exhibit minor heterogeneity. These measuring biases may influence the outcomes of the estimated transmission loss following different boundary condition settings for CFD simulations. In addition, stark differences in flow velocity and vascular shapes among patients may also result in similar differences in the vibroacoustic and hydroacoustic outcomes. Further studies on the differences in flow characteristics among patients are warranted. Differently set boundary conditions for CFD simulations can greatly alter the outcome of interest. Finally, the application of sensors with different specifications and methods of calibration renders the justification of either results of sensing or CFD applications arduous. Therefore, continuous refinement of sensing technologies and boundary condition settings is warranted.

## Conclusion

Flow-induced vibroacoustic and hydroacoustic sounds are two factors that cause PT in patients with temporal dehiscence. According to the outcomes of multi-sensing platforms, venous PT caused by temporal bone dehiscence is the acoustic perception of the sinus flow motion and fluid–structure interaction that transmits to the cochlea predominantly via the air transmission pathway. Nevertheless, the bony tissue, despite its defective appearance as shown by the traditional axial CT scans, remained intact under μCT and SEM examinations. Furthermore, the tight fixation between the surfaces of the sigmoid plate and sigmoid sinus wall, where layers of the dura mater situated in between, may restrict the movement of the vascular wall. This means that vibroacoustic generation of sound is a possibly minor factor causing PT. These findings suggest that multilayer application of sound insulation materials to increase PT transmission loss can be decisive in therapeutic effectiveness. Compared to auricular cartilage–temporalis fascia and gelatin sponge complex repair methods, the combined bone wax–solidified gelatin sponge reconstructive method was the most proficient reconstructive method to preclude both vibroacoustic and hydroacoustic sound transmittance. Although the bulk density of autologous grafts is comparatively high, the lack of thickness (lower planar density) diminishes the volume transmission loss of PT, which is prone to surgical failure. Thus, to maximize the efficacy of transtemporal reconstructive surgery, grafts, e.g., auricular cartilage, and firm synthetic biomaterials such as bone wax or bone cement are encouraged to be applied in a multilayered fashion, achieving sufficient areal density to guarantee adequate sound insulation to resolve PT.

## Data Availability

The original contributions presented in the study are included in the article/Supplementary Material, further inquiries can be directed to the corresponding author.
